# Indoor Residual Spraying in Combination with Insecticide-Treated Nets Compared to Insecticide-Treated Nets Alone for Protection against Malaria: A Cluster Randomised Trial in Tanzania

**DOI:** 10.1371/journal.pmed.1001630

**Published:** 2014-04-15

**Authors:** Philippa A. West, Natacha Protopopoff, Alexandra Wright, Zuhura Kivaju, Robinson Tigererwa, Franklin W. Mosha, William Kisinza, Mark Rowland, Immo Kleinschmidt

**Affiliations:** 1 Department of Infectious Disease Epidemiology, London School of Hygiene & Tropical Medicine, London, United Kingdom; 2 Department of Disease Control, London School of Hygiene & Tropical Medicine, London, United Kingdom; 3 National Institute for Medical Research, Amani Medical Research Centre, Muheza, Tanzania; 4 Muleba District Medical Office, Department of Health, Muleba, Tanzania; 5 Kilimanjaro Christian Medical College, Tumaini University, Moshi, Tanzania; 6 Medical Research Council Tropical Epidemiology Group, London School of Hygiene & Tropical Medicine, London, United Kingdom; University of Oxford, United Kingdom

## Abstract

**Background:**

Insecticide-treated nets (ITNs) and indoor residual spraying (IRS) of houses provide effective malaria transmission control. There is conflicting evidence about whether it is more beneficial to provide both interventions in combination. A cluster randomised controlled trial was conducted to investigate whether the combination provides added protection compared to ITNs alone.

**Methods and Findings:**

In northwest Tanzania, 50 clusters (village areas) were randomly allocated to ITNs only or ITNs and IRS. Dwellings in the ITN+IRS arm were sprayed with two rounds of bendiocarb in 2012. *Plasmodium falciparum* prevalence rate (*Pf*PR) in children 0.5–14 y old (primary outcome) and anaemia in children <5 y old (secondary outcome) were compared between study arms using three cross-sectional household surveys in 2012. Entomological inoculation rate (secondary outcome) was compared between study arms.

IRS coverage was approximately 90%. ITN use ranged from 36% to 50%. In intention-to-treat analysis, mean *Pf*PR was 13% in the ITN+IRS arm and 26% in the ITN only arm, odds ratio = 0.43 (95% CI 0.19–0.97, *n* = 13,146). The strongest effect was observed in the peak transmission season, 6 mo after the first IRS. Subgroup analysis showed that ITN users were additionally protected if their houses were sprayed. Mean monthly entomological inoculation rate was non-significantly lower in the ITN+IRS arm than in the ITN only arm, rate ratio = 0.17 (95% CI 0.03–1.08).

**Conclusions:**

This is the first randomised trial to our knowledge that reports significant added protection from combining IRS and ITNs compared to ITNs alone. The effect is likely to be attributable to IRS providing added protection to ITN users as well as compensating for inadequate ITN use. Policy makers should consider deploying IRS in combination with ITNs to control transmission if local ITN strategies on their own are insufficiently effective. Given the uncertain generalisability of these findings, it would be prudent for malaria control programmes to evaluate the cost-effectiveness of deploying the combination.

**Trial registration:**

www.ClinicalTrials.gov NCT01697852

*Please see later in the article for the Editors' Summary*

## Abstract 2

Philippa West and colleagues compare *Plasmodium falciparum* infection prevalence in children, anemia in young children, and entomological inoculation rate between study arms.

*Please see later in the article for the Editors' Summary*

## Editors' Summary

**Background:**

Every year, more than 200 million cases of malaria occur worldwide, and more than 600,000 people, mainly children living in sub-Saharan Africa, die from this parasitic infection. Malaria parasites, which are transmitted to people through the bites of infected night-flying mosquitoes, cause a characteristic fever that needs to be treated promptly with antimalarial drugs to prevent anaemia (a reduction in red blood cell numbers) and organ damage. Prompt treatment also helps to reduce malaria transmission, but the mainstays of global malaria control efforts are the provision of insecticide-treated nets (ITNs) for people to sleep under to avoid mosquito bites, and indoor residual spraying (IRS) of houses with insecticides, which prevents mosquitoes from resting in houses. Both approaches have been scaled up in the past decade. About 54% of households in Africa now own at least one ITN, and 8% of at-risk populations are protected by IRS. As a result of the widespread deployment of these preventative tools and the increased availability of effective antimalarial drugs, malaria-related deaths in Africa fell by 45% between 2000 and 2012.

**Why Was This Study Done?:**

Some countries have chosen to use ITNs and IRS in combination, reasoning that this will increase the proportion of individuals who are protected by at least one intervention and may provide additional protection to people using both interventions rather than one alone. However, providing both interventions is costly, so it is important to know whether this rationale is correct. In this cluster randomised controlled trial (a study that compares outcomes of groups of people randomly assigned to receive different interventions) undertaken in the Muleba District of Tanzania during 2012, the researchers investigate whether ITNs plus IRS provide more protection against malaria than ITNs alone. Malaria transmission occurs throughout the year in Muleba District but peaks after the October–December and March–May rains. Ninety-one percent of the district's households own at least one ITN, and 58% of households own enough ITNs to cover all their sleeping places. Annual rounds of IRS have been conducted in the region since 2007.

**What Did the Researchers Do and Find?:**

The researchers allocated 50 communities to the ITN intervention or to the ITN+IRS intervention. Dwellings allocated to ITN+IRS were sprayed with insecticide just before each of the malaria transmission peaks in 2012. The researchers used household surveys to collect information about ITN coverage in the study population, the proportion of children aged 0.5–14 years infected with the malaria parasite *Plasmodium falciparum* (the prevalence of infection), and the proportion of children under five years old with anaemia. IRS coverage in the ITN+IRS arm was approximately 90%, and 50% of the children in both intervention arms used ITNs at the start of the trial, declining to 36% at the end of the study. In an intention-to-treat analysis (which assumed that all study participants got the planned intervention), the average prevalence of infection was 13% in the ITN+IRS arm and 26% in the ITN arm. A per-protocol analysis (which considered data only from participants who received their allocated intervention) indicated that the combined intervention had a statistically significant protective effect on the prevalence of infection compared to ITNs alone (an effect that is unlikely to have arisen by chance). Finally, the proportion of young children with anaemia was lower in the ITN+IRS arm than in the ITN arm, but this effect was not statistically significant.

**What Do These Findings Mean?:**

These findings provide evidence that IRS, when used in combination with ITNs, can provide better protection against malaria infection than ITNs used alone. This effect is likely to be the result of IRS providing added protection to ITN users as well as compensating for inadequate ITN use. The findings also suggest that the combination of interventions may reduce the prevalence of anaemia better than ITNs alone, but this result needs to be confirmed. Additional trials are also needed to investigate whether ITN+IRS compared to ITN reduces clinical cases of malaria, and whether similar effects are seen in other settings. Moreover, the cost-effectiveness of ITN+IRS and ITN alone needs to be compared. For now, though, these findings suggest that national malaria control programs should consider implementing IRS in combination with ITNs if local ITN strategies alone are insufficiently effective and cannot be improved.

**Additional Information:**

Please access these websites via the online version of this summary at http://dx.doi.org/10.1371/journal.pmed.1001630.

- Information is available from the World Health Organization on malaria (in several languages), including information on insecticide-treated bed nets and indoor residual spraying; the *World Malaria Report 2013* provides details of the current global malaria situation
- The US Centers for Disease Control and Prevention provides information on malaria, on insecticide-treated bed nets, and on indoor residual spraying; it also provides a selection of personal stories about malaria
- Information is available from the Roll Back Malaria Partnership on the global control of malaria and on the Global Malaria Action Plan (in English and French); its website includes fact sheets about malaria in Africa and about nets and insecticides
- MedlinePlus provides links to additional information on malaria (in English and Spanish)

## Introduction

In the past decade, insecticide-treated net (ITN) distribution has been scaled up across Africa in line with the Abuja Declaration in 2000 [1]. The percentage of households that owned at least one ITN in Africa increased from 3% in 2000 to 54% in 2013. The World Health Organization (WHO) policy that ITNs should be provided to everyone in malaria risk areas (universal coverage) [2] has been adopted by 34 of the 44 malaria endemic countries in Africa [3]. Indoor residual spraying (IRS) of houses, the second major vector control tool used to prevent malaria, has similarly been scaled up. The proportion of at-risk populations protected by IRS increased from less than 5% in 2005 to 8% in 2012 [3]. As a result of the increase in the deployment of these preventive tools and the increased availability and use of artemisinin-based combination therapies, malaria-related mortality fell by 45% between 2000 and 2012 in Africa, but there remained an estimated 165 million cases and 562,000 deaths due to malaria in 2012 [3].

In an attempt to reduce the malaria burden further, a number of countries have chosen to use ITNs and IRS in combination. Fifty-seven countries, 31 of which are in Africa, use both IRS and ITNs, in at least some areas [3]. Applying ITNs and IRS in the same area can increase the proportion of individuals who are protected by at least one intervention or, more optimally, may provide additional protection for those protected by both interventions compared to those receiving one method alone [4]–[7].

Since the cost of implementing both IRS and universal coverage of ITNs is much greater than the cost of implementing only one of the interventions [8], it is important to know what extra protection is gained by adding a second intervention, to help national malaria control programmes and international funding agencies such as the President's Malaria Initiative (PMI) and the Global Fund to Fight AIDS, Tuberculosis and Malaria make decisions that are based on evidence of likely impacts and costs. This is particularly significant now, since it is estimated that global funding for malaria is less than half of what is needed to attain universal coverage of malaria vector control, i.e., access to either ITNs or IRS [9].

It is unclear from current evidence whether combined use of ITNs and IRS provides an additional benefit compared to using either intervention alone, and whether this will be similar across transmission settings [4]–[7],[10],[11]. A recent trial in Benin found no added benefit to using IRS in combination with ITNs compared to ITNs alone [10]. However, this trial had a relatively small sample size, and its findings may be applicable to only a particular transmission setting in west Africa [12].

To help define future malaria control policy in Africa, the PMI decided to sponsor an independent two-arm cluster randomised controlled trial (CRT) to compare the protective effectiveness of IRS in combination with high coverage of ITNs with high coverage of ITNs alone for malaria transmission control.

Tanzania has a high malaria disease burden, with a national average of 9% of children under 5 y being infected with malaria parasites [13]. Malaria control activities have been scaled up nationally since 2005 [14]–[16]. A universal coverage campaign (UCC) primarily funded by the Global Fund to Fight AIDS, Tuberculosis and Malaria distributed long-lasting insecticidal nets (LLINs) free of charge in 2011 to top up coverage from previous distributions [14],[15],[17]. IRS, funded by the PMI, commenced in 2007 in two districts of Kagera Region, in northwest Tanzania, and has since been extended to cover 18 districts [18]. Because IRS is costly and logistically intensive [8],[19], there is an urgent need to know whether it is necessary to continue with IRS after an ITN UCC has been successfully completed.

The trial was carried out in 109 rural villages in Muleba District (1°45′S 31°40′E), Kagera Region [20],[21]. The study area includes 68,108 households at an altitude ranging from 1,100 to 1,600 m above sea level. Rainfall occurs in two seasons: the “short rains” in October–December (average monthly rainfall 160 mm) and the “long rains” in March–May (average monthly rainfall 300 mm) [22], with malaria transmission occurring throughout the year and peaking after the rainy seasons [23]. Annual rounds of IRS with the pyrethroid lambda-cyhalothrin (ICON 10CS, Syngenta) were conducted between 2007 and 2011 in Muleba District, i.e., in the entire study area. The predominant malaria vectors are *Anopheles gambiae* s.s. and *An. arabiensis* [24]. Tests of mosquito susceptibility using standard WHO bioassays showed resistance to pyrethroids in *An. gambiae* s.s. in 2011 [24]. As a result, IRS policy was changed to use the carbamate insecticide bendiocarb (Ficam 80% wettable powder, Bayer) by the PMI in 2012.

## Methods

### Ethics and Community Sensitisation

The trial was approved by the ethics review committees of the Kilimanjaro Christian Medical College, the Tanzanian National Institute for Medical Research, and the London School of Hygiene and Tropical Medicine. Written informed consent was obtained from all respondents. Prior to the baseline surveys, village and hamlet leaders were invited to sensitisation sessions conducted by district health officers.

The trial was registered with ClinicalTrials.gov (registration number NCT01697852) in September 2012. The trial was not registered earlier because the authors were not aware of journal requirements for prospective registration. All authors have affirmed that any trials they are involved in on the same or a related drug or intervention are registered. An accurate summary of the trial's results has been submitted to ClinicalTrials.gov.

### Study Design

A CRT was conducted, comparing the *Plasmodium falciparum* prevalence rate (*Pf*PR) in children 0.5–14 y old between communities targeted to receive both high-coverage IRS and high coverage of ITNs (ITN+IRS arm) and communities targeted for high coverage of ITNs only (standard-care control arm). Secondary outcomes were moderate/severe anaemia (haemoglobin <8 g/dl) in children under 5 y old and entomological inoculation rate (EIR) due to *An. gambiae* s.l.

Power calculations showed that 25 clusters per study arm were required, with 80 children per cluster, to give 80% power to detect a true absolute difference in *Pf*PR of at least 3% between study arms (relative difference 31%) with 5% significance (two-sided), based on an expected prevalence in the ITN only arm of 9% (*Pf*PR in first baseline survey). The between-cluster coefficient of variation (*k*) was calculated as 0.25 from the first pre-randomisation baseline survey [25].

Each cluster consisted of at least one village and was divided into a core surveillance area consisting of at least 200 houses and approximately 1 km radius, where the surveys were conducted, and an outer buffer zone, 1 km in width, which also received the allocated treatment but in which no outcome monitoring was done. Villages were eligible for inclusion in the study if they were within daily commuting distance for survey work and had been sprayed with IRS in the baseline year.

All clusters received LLINs from the UCC in 2011. Twenty-five clusters were randomly allocated to receive IRS, in addition to ITNs, using restricted randomisation to limit potential imbalance between study arms [25]. Baseline surveys provided data on seven criteria for which the study arms were balanced by constraining the randomisation (Table 1). 200,000 random allocations were generated. Mean values for each arm were calculated from cluster summaries for each of the seven restriction variables; 25,119 randomisations fulfilled the restriction criteria and were therefore eligible. These allocations were tested for independence between any two clusters. The large number of acceptable allocations, of which one was randomly selected, ensured that the restriction did not affect the validity of inference. There was no evidence of dependence between any pair of clusters [25],[26].

**Table 1:** Restriction variables for randomisation and realisation of balance between the study arms.

Variable	Maximum Difference in Means between Study Arms^a^	ITN Arm^a^	ITN+IRS Arm^a^	Actual Difference
*Pf*PR^b^ in February–March 2011^c^	3%	9.9%	9.3%	0.5%
*Pf*PR in June–July 2011^d^	3%	22.4%	19.6%	2.7%
Housing density^e^	20 HH/km^2^	165.1 HH/km^2^	152.6 HH/km^2^	12.5 HH/km^2^
Mean elevation	50 m	1,364.8 m	1,330.7 m	34.1 m
ITN usage^d^^,^ f	5%	35.0%	30.4%	4.6%
Adequate LLIN ownership^e^^,^ g	5%	61.3%	56.3%	5.0%
Clusters with entomological surveillance	Count of 2	20 clusters	20 clusters	0 clusters

a Means for each study arm were calculated from cluster summaries.

b *Pf*PR from RDTs.

c Recorded in baseline survey 1(February–March 2011).

d Recorded in baseline survey 2 (June–July 2011) after the UCC.

e Housing density in surveillance area of clusters.

f Net used the night before the survey in all age groups.

g Percentage of households with at least one LLIN per two people.

HH, household.

### Interventions

Households in the study area with children aged under 5 y received LLINs from a national distribution campaign in 2009 [16]. In 2011, the district health authority, supported by Mennonite Economic Development Associates, completed a UCC that distributed 144,000 LLINs (Olyset, Sumitomo Chemicals) to the population of Muleba District, including all study clusters. The campaign aimed to top up net coverage, so that every sleeping place had one ITN. After the UCC, 91% of households owned at least one ITN, and 58% of households owned enough ITNs to cover all their sleeping places [20].

Spraying was conducted by RTI International on behalf of PMI in the ITN+IRS study arm. The interior walls of each dwelling were sprayed with the carbamate insecticide bendiocarb (Ficam 80% wettable powder, Bayer) at 400 mg/m^2^ between December 2011 and January 2012 (round 1), and between April and May 2012 (round 2). Spray rounds were timed to precede the peak in malaria cases that normally occurs at the end of each rainy season, taking into account the relatively short residual duration of bendiocarb.

Bendiocarb is a carbamate insecticide recommended by WHO for IRS [27],[28]. It is one of the few insecticides evaluated and approved by the WHO Pesticide Evaluation Scheme that has the potential to control pyrethroid-resistant mosquitoes, is odour-free, and is safe to house occupants at the recommended application rate [27]. Before obtaining WHO approval, all IRS insecticides are subject to risk assessment by WHO toxicologists [29]. Bendiocarb is an acetylcholinesterase inhibitor, but no serious adverse effects due to bendiocarb IRS have been reported in the recent medical literature.

### Surveys

**Figure 1:**
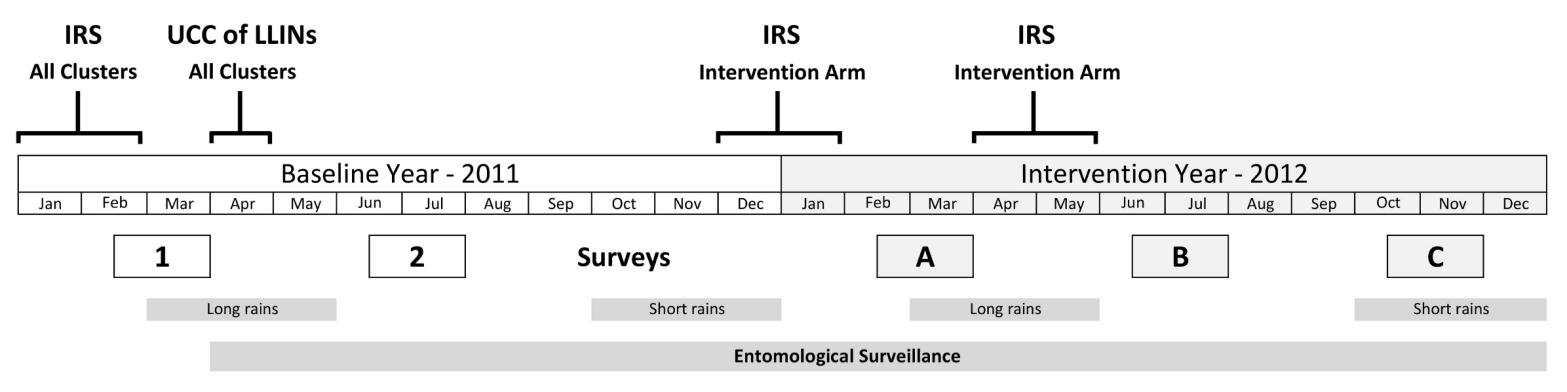
Study timetable. Surveys 1 and 2 are baseline surveys. Surveys A, B, and C are post-intervention.

Three post-intervention cross-sectional household surveys were undertaken in 2012 (see Figure 1). Survey A (23 February–31 March) was after the short rainy season and 2 mo after the first spray round. Survey B (25 June–31 July) was after the long rainy season, 6 mo after the first spray round, and 2 mo after the second spray round. Survey C (25 October–4 December) was 6 mo after the second spray round and 10 mo after the first. Baseline surveys were conducted in 2011 during the same periods as surveys A and B.

For each survey, 80 households were randomly selected in the core area of each cluster. Households were eligible for the study if they had children aged 0.5–14 y. Any child aged 0.5–14 y was eligible to be included in the study. Up to three children per household were randomly selected for testing. Allowing for ineligible households, absence on the day of the survey, and refusals at the household and individual level, it was estimated that this would provide on average 80 children for testing per cluster.

The household head or another responsible adult from the household was interviewed, after seeking written informed consent. Data on IRS coverage, bed net ownership and usage, demographics of household members, and other household characteristics were gathered using an adapted version of the standard Malaria Indicator Survey [30].

Selected children were tested on the following day for malaria parasites using a rapid diagnostic test (RDT) (CareStart [Pan] Malaria, DiaSys) and had haemoglobin levels measured using HemoCue Hb 201^+^ (Aktiebolaget Leo Diagnostics). Individuals testing positive by RDT were treated with artemether/lumefantrine (Artefan 20/120, Ajanta Pharma) following national treatment guidelines.

Entomological surveillance was carried out in the core surveillance areas of a subset of 40 of the 50 clusters from April 2011 to December 2012. For one night of each month US Centers for Disease Control and Prevention light traps for mosquito collections were set up in eight randomly selected houses in each cluster (320 houses per month). Anopheles mosquitoes collected were identified to species using a simplified morphological key adapted from Gillies and Coetzee [31]. A sub-sample of *An. gambiae* s.l. individuals were tested using real-time PCR TaqMan assay to distinguish between the two sibling species *An. gambiae* s.s. and *An. arabiensis* [32]. Mosquitoes were also tested for *P. falciparum* sporozoites (*P. falciparum* circumsporozoite protein) using ELISA [33].

### Statistical Analysis

Statistical analysis was done in Stata 12 (Statacorp) and R version 2.13.1 (R Foundation for Statistical Computing). The odds of *Pf*PR and moderate/severe anaemia for individuals were compared between study arms in intention-to-treat (ITT) analysis using logistic regression. Mean haemoglobin was compared between the study arms using linear regression. A robust variance estimator was used to calculate standard errors to adjust for within-cluster correlation of responses (Stata survey commands, first-order Taylor-series linearization method) [34],[35]. *Pf*PR was considered as *P. falciparum* alone or mixed infections as detected by the RDT. The overall odds ratio (OR) for the three surveys combined was calculated accounting for survey. An adjusted Wald test was performed to test whether there was evidence for effect modification between study arm and survey round. A sensitivity analysis was conducted excluding one cluster from the ITN only arm that mistakenly received IRS, to assess the impact of this protocol violation on the results of ITT analysis. Because of the wide variation in cluster-level estimates of *Pf*PR at baseline, an OR for ITN+IRS versus ITN alone was calculated adjusting for baseline *Pf*PR.

A secondary per-protocol analysis was performed, in which individuals from the ITN+IRS arm who used an ITN and lived in a house sprayed in the most recent round of IRS were compared to individuals who used an ITN in the ITN only arm. The cluster that violated the protocol was excluded from the per-protocol analysis.

The monthly EIR was calculated as the daily EIR found during the one night collection multiplied by the number of days in the month. Mean EIRs were compared between study arms using negative binomial regression and adjusting for within-cluster correlation.

## Results

At baseline, *Pf*PR, anaemia, ITN ownership, ITN usage, and mean EIR per month (Table 2) were similar in the two study arms. *Pf*PR in children aged 6 mo to 14 y old was 9.3% (95% CI 5.9%–14.5%) after the short rains (survey A, February–March) and 22.8% (95% CI 17.3%–29.4%) after the long rains (survey B, June–July). Anaemia in children 0.5–4 y was 6.2% (95% CI 4.5%–8.5%) after the long rains.

**Table 2:** Baseline characteristics of individuals and households by study arm, Muleba District, 2011.

Characteristic	ITN Only ArmPercent [95% CI] (*n*)	ITN+IRS ArmPercent [95% CI] (*n*)
*Pf*PR in March 2011^a^^,^ b ^,^ c	10.3 [5.2–19.3] (2,487)	8.4 [4.5–15.3] (2,655)
*Pf*PR in July 2011^a^^,^ b ^,^ d	24.6 [17.0–34.3] (2,121)	21.0 [13.8–30.5] (2,185)
Moderate/severe anaemia^a^^,^ d ^,^ e	6.4 [3.9–10.2] (785)	6.1 [4.1–8.9] (841)
Mean haemoglobin (g/dl)^a^^,^ d ^,^	10.6 [10.4–10.9] (785)	10.6 [10.4–10.9] (841)
ITN use in all age groups^a^^,^ d ^,^ f	53.3 [48.2–58.3] (6,755)	58.2 [53.8–62.5] (6,913)
Households with adequate ITNs^d^^,^ g ^,^ h	54.5 [49.5–59.5] (1,243)	62.3 [57.3–67.1] (1,250)
Households with ≥1 ITN^d^^,^ g	88.9 [86.0–91.3] (1,248)	92.6 [90.8–94.0] (1,251)
Households received IRS in 2011^c^^,^ g ^,^ i	94.4 [91.3–96.5] (1,598)	95.5 [93.5–96.9] (1,640)
Mean *An. gambiae* mosquitoes per house per night^g^^,^ j	3.1 [1.0–9.6] (1,055)	2.2 [0.5–9.1] (1,120)
Sporozoite rate^a^^,^ k	1.1 [0.8–1.4] (1,359)	2.0 [1.4–2.8] (1,466)
Mean EIR/month^l^	1.1 [0.4–2.8]	1.3 [0.4–4.4]

a Calculated from individual-level data.

b *Pf*PR from RDTs.

c Recorded in baseline survey 1 (February–March 2011).

d Baseline survey 2 (June–July 2011) after the UCC.

e Haemoglobin <8 g/dl.

f Reported sleeping under an ITN the night previous to the survey.

g Calculated from household-level data.

h At least one ITN per sleeping place.

i Approximately 1 mo after spraying.

j Arithmetic mean.

k Proportion of mosquitoes positive for *P. falciparum* sporozoites.

l Number of infective bites per month.

**Figure 2:**
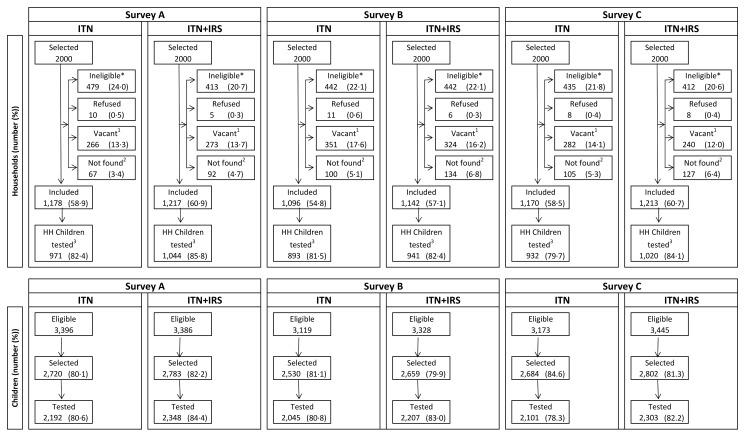
Trial profile for study households and children in the ITN only and ITN+IRS study arms. Survey A = 2 mo after first intervention spray. Survey B = 6 mo after first intervention spray and 2 mo after second intervention spray. Survey C = 10 mo after first intervention spray and 6 mo after second intervention spray. *No children 0.5–14 y old. ^1^Dwelling vacant for survey duration. ^2^Includes not found (91.0%), not visited (2.4%), and missing data (6.6%). ^3^Households (HH) that were included and where children attended for testing.

Of the 2,000 houses selected in each study arm for each post-intervention survey, 20% to 24% had no children between 0.5 and 14 y old (were ineligible), 13% to 18% were vacant on the day of survey, fewer than 1% refused to participate, and 55% to 61% participated in the survey (Figure 2). Of the children selected for RDT, 81%–84% were tested. Post-intervention IRS coverage reported by householders was 92.1% after the first spray round and 89.5% after the second (Table 3).

**Table 3:** IRS coverage, ITN ownership, and ITN usage in the intervention year, Muleba District, 2012.

Survey	Arm	Reported IRS Coverage^a^Percent [95% CI] (*n* b)	Adequate ITN Ownership^c^Percent [95% CI] (*n* b)	≥1 ITN Owned^d^Percent [95% CI] (*n* b)	ITN Use^e^Percent [95% CI] (*n* f)
**Survey A**	ITN only	3.3 [1.8–5.9] (1,177)	52.2 [47.8–56.5] (1,178)	85.8 [83.7–87.7] (1,177)	46.6 [41.7–51.6] (2,193)
	ITN+IRS	92.1 [88.4–94.7] (1,215)	57.2 [53.6–60.7] (1,215)	89.0 [87.1–90.6] (1,216)	53.0 [47.5–58.3] (2,349)
**Survey B**	ITN only	5.2 [1.3–18.6] (1,094)	51.6 [47.0–56.0] (1,094)	82.5 [78.7–85.7] (1,096)	40.7 [34.7–47.0] (2,045)
	ITN+IRS	89.5 [84.0–93.2] (1,138)	57.4 [54.0–60.9] (1,142)	88.2 [85.7–90.3] (1,142)	44.1 [39.2–49.2] (2,207)
**Survey C**	ITN only	13.0 [6.6–24.1] (1,165)	52.8 [47.6–58.0] (1,168)	78.2 [74.3–81.6] (1,170)	36.0 [29.8–42.6] (2,101)
	ITN+IRS	89.3 [83.6–93.2] (1,209)	56.8 [51.7–61.8] (1,211)	83.8 [79.9–87.1] (1,211)	36.1 [31.0–41.5] (2,303)

Survey A = 2 mo after first intervention spray. Survey B = 6 mo after first intervention spray and 2 mo after second intervention spray. Survey C = 10 mo after first intervention spray and 6 mo after second intervention spray.

a Reported spray status of household in the spray round preceding the survey.

b Households.

c Percentage of households with sufficient ITNs for at least one per sleeping place.

d Percentage of households with at least one ITN.

e Percentage of study children that reported sleeping under an ITN the night previous to the survey. ITN usage in all age groups was very similar to ITN use in the study children.

f Individuals.

In the intervention year, the percentage of houses with sufficient ITNs for each sleeping place remained stable over successive surveys and was similar between study arms (range 52%–57%; Table 3). 82.2% and 87.0% of households owned at least one ITN in the ITN only arm and the ITN+IRS arm, respectively (all surveys combined), with weak evidence that the percentage of households that owned at least one ITN was lower in the ITN only arm, and that it decreased from survey A to survey C in both arms (Table 3). ITN usage in children was similar between study arms but declined from 50% in survey A to 36% in survey C.

The primary outcome *Pf*PR was lower in the ITN+IRS arm than in the ITN only arm in all three surveys in the intervention year (Table 4). For all three surveys combined, the overall OR was 0.43 (95% CI 0.19–0.97), with weak evidence that the intervention effect differed between surveys (interaction *p = *0.08). The strongest effect was observed in survey B (OR 0.33, 95% CI 0.15–0.75), which was conducted at the peak of malaria transmission after the long rains, 6 mo after the first IRS and 2 mo after the second IRS. The evidence for an effect was weaker in survey A (OR 0.51, 95% CI 0.24–1.09), conducted shortly after the first IRS round, and in survey C (OR 0.48, 95% CI 0.18–1.24), conducted several months after the main transmission season and 6 mo after last spray round. The range of cluster-specific estimates for *Pf*PR was 0% to 92% in the ITN only arm and 0% to 68% in the ITN+IRS arm. The sensitivity analysis showed that excluding the cluster from the ITN only arm that had received IRS did not affect the results of the ITT analysis (Table S1). The overall OR for all three surveys combined was very similar after adjusting for baseline *Pf*PR, OR = 0.41, but the precision of the estimate was increased (95% CI 0.29–0.59, *p<*0.0001).

**Table 4:** *Pf*PR in children 0.5–14 y old in the ITN only and ITN+IRS arms (intention to treat) in survey A, B, and C, Muleba District, Tanzania, 2012.

Survey	Arm	*Pf*PR^a^Percent [95% CI] (*n*)	OR [95% CI], *p-*Value
**Survey A**	ITN only	23.6 [15.4–34.2] (2,191)	1.00
	ITN+IRS	13.6 [8.3–21.4] (2,342)	0.51 [0.24–1.09], *p = *0.082
**Survey B**	ITN only	30.5 [20.2–43.4] (2,033)	1.00
	ITN+IRS	12.7 [7.4–21.0] (2,204)	0.33 [0.15–0.75], *p = *0.009
**Survey C**	ITN only	24.5 [14.2–38.9] (2,091)	1.00
	ITN+IRS	13.4 [7.3–23.4] (2,285)	0.48 [0.18–1.24], *p = *0.127
**All three surveys combined**	ITN only	26.1 [16.7–38.4] (6,315)	1.00
	ITN+IRS	13.3 [7.9–21.5] (6,831)	0.43 [0.19–0.97], *p = *0.043^b^

Survey A = 2 mo after first intervention spray. Survey B = 6 mo after first intervention spray and 2 mo after second intervention spray. Survey C = 10 mo after first intervention spray and 6 mo after second intervention spray.

a *Pf*PR from RDTs.

b Adjusted for survey.

Prevalence of moderate to severe anaemia in children under 5 y old, a secondary outcome, was lower in the ITN+IRS arm in all post-intervention surveys, but the difference was statistically significant only in survey B (Table 5). Mean haemoglobin was higher in children under 5 y old in the ITN+IRS arm than in the ITN only arm in all three surveys. The evidence for an effect was greatest in survey B (0.49 g/dl, 95% CI 0.10–0.89, *p = *0.016), with a non-significant result in survey A (0.28 g/dl, 95% CI −0.02 to 0.59, *p = *0.065) and survey C (0.36 g/dl, 95% CI −0.02 to 0.73, *p = *0.060).

**Table 5:** Anaemia and mean haemoglobin in children under 5+IRS arms (intention to treat), for survey A, B, and C, Muleba District, Tanzania, 2012.

Survey	Arm	Anaemia Prevalence^a^		Mean Haemoglobin (g/dl)	
		Percent [95% CI] (*n*)	OR [95% CI], *p-*Value	Mean [95% CI] (*n*)	Difference [95% CI], *p-*Value
**Survey A**	ITN only	6.0 [4.1–8.7] (815)	1.00	10.6 [10.4–10.8] (815)	
	ITN+IRS	3.9 [2.5–6.2] (864)	0.64 [0.34–1.19], *p = *0.155	10.9 [10.7–11.1] (864)	0.28 [−0.02 to 0.59], *p = *0.065
**Survey B**	ITN only	4.7 [2.6–8.6] (737)	1.00	10.9 [10.6–11.2] (737)	
	ITN+IRS	2.2 [1.3–3.6] (784)	0.44 [0.20–1.01], *p = *0.053	11.4 [11.2–11.6] (784)	0.49 [0.10 to 0.89], *p = *0.016
**Survey C**	ITN only	3.2 [1.8–5.7] (739)	1.00	10.8 [10.6–11.1] (739)	
	ITN+IRS	2.6 [1.6–4.4] (831)	0.81 [0.37–1.77], *p = *0.590	11.2 [11.0–11.4] (831)	0.36 [−0.02 to 0.73], *p = *0.060
**All three surveys combined**	ITN only	4.7 [3.2–6.9] (2,291)	1.00	10.8 [10.5–11.0] (2,291)	
	ITN+IRS	2.9 [2.0–4.3] (2,479)	0.62 [0.34–1.10], *p = *0.102^b^	11.2 [11.0–11.3] (2,479)	0.37 [0.07 to 0.68], *p = *0.017^b^

Survey A = 2 mo after first intervention spray. Survey B = 6 mo after first intervention spray and 2 mo after second intervention spray. Survey C = 10 mo after first intervention spray and 6 mo after second intervention spray.

a Prevalence of moderate/severe anaemia (haemoglobin <8 g/dl).

b Adjusted for survey.

Mean EIR per month, a secondary outcome, was 0.22 in the ITN+IRS arm and 1.26 in the ITN only arm (rate ratio = 0.17, 95% CI 0.03–1.08, *p = *0.059; Table 6).

**Table 6:** Mean number of *An. gambiae* mosquitoes per household, sporozoite rate, and EIR in the ITN only and ITN+IRS arms during the post-intervention period, Muleba District, Tanzania, 2011–2012.

Arm	Mean or Percent [95% CI] (*n*)^a^	Effect [95% CI], *p*-Value
**Mean** b ***An. gambiae*** ** per house per night**		
ITN only	1.7 [0.5–6.4] (1,892)	
ITN+IRS	0.4 [0.1–1.4] (1,893)	Rate ratio = 0.23 [0.04–1.44], *p = *0.113
**Sporozoite rate** c		
ITN only	2.5 [2.1–3.1] (3,059)	
ITN+IRS	1.8 [0.5–6.2] (717)	OR = 0.72 [0.21–2.53], *p = *0.600
**Mean EIR/month** d		
ITN only	1.3 [0.3–4.6]	
ITN+IRS	0.2 [0.1–0.8]	Rate ratio = 0.17 [0.03–1.08], *p = *0.059

a Data are mean [95% CI] (number of houses) for mean *An. gambiae* per house per night and percent [95% CI] (number of *An. gambiae*) for sporozoite rate.

b Arithmetic mean.

c Proportion of mosquitoes positive for *P. falciparum* sporozoites.

d Number of infective bites per month.

The between-cluster coefficient of variation (*k*) was 0.20, 0.28, and 0.26 in the three post-intervention surveys, respectively. For each survey, *k* was similar in the two arms.

For all surveys, per-protocol analysis showed statistically significant evidence for a protective effect of the combined intervention on *Pf*PR (survey A: OR 0.39, 95% CI 0.18–0.81; survey B: OR 0.21, 95% CI 0.09–0.49; and survey C: OR 0.27, 95% CI 0.10–0.73; Table 7).

**Table 7:** Per-protocol analysis of *Pf*PR in children 0.5–14 y old and anaemia in children under 5 y old in surveys A, B, and C.

Survey	Arm	PrevalencePercent [95% CI] (*n*)	OR [95% CI], *p-*Value
***Pf*** **PR** a			
Survey A	ITN^b^	26.7 [17.5–38.6] (954)	1.00
	ITN+IRS^c^	12.3 [7.8–18.9] (1,142)	0.39 [0.18–0.81], *p = *0.013
Survey B	ITN^b^	35.5 [23.2–50.2] (782)	1.00
	ITN+IRS^c^	10.2 [5.7–17.7] (892)	0.21 [0.09–0.49], *p = *0.001
Survey C	ITN^b^	29.4 [16.7–46.4] (707)	1.00
	ITN+IRS^c^	10.1 [5.4–18.2] (770)	0.27 [0.10–0.73], *p = *0.011
**Anaemia** d			
Survey A	ITN^b^	5.9 [3.5–9.7] (390)	1.00
	ITN+IRS^c^	3.8 [1.8–7.5] (453)	0.62 [0.25–1.55], *p = *0.301
Survey B	ITN^b^	5.4 [2.2–12.5] (295)	1.00
	ITN+IRS^c^	1.9 [0.8–4.1] (374)	0.33 [0.10–1.12], *p = *0.076
Survey C	ITN^b^	4.0 [2.2–7.0] (303)	1.00
	ITN+IRS^c^	2.3 [1.0–5.0] (305)	0.57 [0.21–1.55], *p = *0.264

Muleba, Tanzania, 2012; analysis restricted to ITN users in both study arms. Survey A = 2 mo after first intervention spray. Survey B = 6 mo after first intervention spray and 2 mo after second intervention spray. Survey C = 10 mo after first intervention spray and 6 mo after second intervention spray.

a *Pf*PR from RDTs.

b ITN used by the individual the night preceding the survey in the ITN only arm.

c ITN used by the individual the night preceding the survey, and household with IRS in the ITN+IRS arm. One cluster that was allocated to be in the ITN only arm but received IRS in the second spray round was excluded from this analysis.

d Prevalence of moderate/severe anaemia (haemoglobin <8 g/dl).

## Discussion

This is the first randomised trial to our knowledge that provides evidence that IRS, when used in combination with ITNs, can give significant added protection against malarial infection compared to ITN use alone. There was also some evidence that anaemia prevalence was lower in communities with the combination. Exposure to infectious mosquito bites was about one-sixth in communities with the combined intervention compared to those in the ITN only arm. Two rounds of IRS with bendiocarb were conducted to overcome the short residual activity of the insecticide [27],[36] and to ensure that there was active ingredient on the walls of sprayed homes throughout the transmission season.

IRS coverage in the ITN+IRS arm was high at approximately 90% in both spray rounds, which would have optimised its effectiveness [37]. On the other hand, whilst 85% of households owned at least one ITN, use of ITNs was modest, declining to 36% by the end of the study. The low usage of ITNs means that the addition of IRS may have simply protected those who were not using an ITN, thus compensating for low ITN usage rather than offering additional protection to net users. This interpretation is contradicted by the results of a per-protocol analysis, which excluded those not using ITNs, showing strong evidence that ITN users whose houses were sprayed were additionally protected by IRS. The estimated reduction in *Pf*PR associated with the combination of interventions was greater in the per-protocol analysis than in the ITT analysis in each survey. Per-protocol analysis excludes non-compliers (for IRS and ITN) and therefore may have been influenced by confounders. It is likely that the observed overall effect of the intervention combination was a result of both IRS protecting those not using ITNs, and IRS additionally protecting ITN users.

A potential negative impact of the combination of interventions is that having their house sprayed may encourage some residents to stop sleeping under an ITN. This was not observed in this study; ITN usage was similar between the villages with and without IRS in each post-intervention survey.

ITN usage and ownership was slightly higher at baseline in the ITN+IRS arm compared to the ITN only arm, but the 95% confidence intervals for these estimates overlapped. This non-significant difference could have led to a slight overestimation of the effect size. *Pf*PR was slightly lower at baseline in the ITN+IRS arm compared to the ITN only arm, but the effect size did not change after adjusting for *Pf*PR at baseline. This suggests that baseline *Pf*PR was not confounding the relationship between study arm and *Pf*PR (the outcome). In the baseline year, malaria prevalence was higher in June–July after the long rainy season than in February–March after the short rains. In the intervention year, the prevalence similarly increased in June–July (survey B) in the ITN only arm, but prevalence in the ITN+IRS arm remained low, suggesting IRS and ITNs in combination prevented the seasonal increase in infections.

The added protective effect of IRS peaked in the second survey, at the height of transmission after the long rains. This was probably the optimal time for the insecticide to reduce the abundance of the mosquito population (N. Protopopoff, personal communication) and thus to observe the impact of IRS on the prevalence of malarial infections. The limited residual activity of bendiocarb IRS has been shown to reduce its protective effectiveness 3–5 mo after spraying, which probably accounts for the loss of added benefit seen in the third survey, which was 6 mo after the last spray round at the beginning of the short rains [27],[36]. Implementing IRS with long-lasting insecticide formulations might be necessary to maintain the effectiveness of the combination throughout the year. Alternatively, the time between IRS rounds could be reduced, but this would considerably raise the cost of the combined intervention [38].

The secondary outcomes anaemia and EIR also pointed to added protection being provided by the combination of IRS and ITNs, but the evidence for these endpoints was weaker. The combination intervention was associated with higher haemoglobin levels in children under 5 y, particularly at the peak of the transmission season. The study had been powered to show a difference in the primary outcome (*Pf*PR), and therefore may have been underpowered for these secondary outcomes. Nevertheless, the results for all outcomes are consistent.

One of the limitations of this study is that clinical incidence of malaria could not be recorded in addition to infection prevalence because recording of confirmed malaria cases was unreliable because of stock-outs of RDTs at health facilities. Implementing both IRS and universal coverage of ITNs is obviously considerably more costly than ITNs alone. Estimating the cost-effectiveness of the combination compared to ITNs alone was beyond the scope of this particular research. Although IRS is known to be highly cost-effective [8],[39]–[43], the marginal cost per case averted through using IRS in combination with ITNs should ideally be assessed in future studies. This is particularly important in light of the funding gap that has been identified for meeting the demand for universal coverage of vector control for populations in malaria endemic regions [3].

Previous studies have investigated the combined use of multiple vector control methods versus one method alone, but the results have been inconsistent [4],[44]–[47]. The only published trial data are from a 28-cluster, four-arm CRT carried out in Benin that compared (1) targeted coverage of LLINs (pregnant women and children only), (2) universal coverage of LLINs, (3) targeted coverage of LLINs combined with bendiocarb IRS, and (4) universal coverage of LLINs combined with bendiocarb-treated wall linings [10]. The study found no difference in malaria incidence, geometric mean parasite density, or mosquito abundance between any of the study arms. The lack of any evidence of an added benefit of the combined interventions over the use of LLINs alone has to be viewed against the modest sample size, and hence potentially low power of this trial [12], and the lack of a comparator arm with universal coverage of ITNs.

There are a number of differences between the Benin trial and the current study that may have contributed to the discordant results. In the Benin trial, the interval between IRS rounds was 8 mo, whereas it was only 4 mo in the current study, as IRS was timed according to the seasonal peaks in cases, and taking account of its short residual duration on walls. The first two cross-sectional surveys for the current trial were timed to coincide with the seasonal peaks in cases and were only 2 mo after each IRS round, whereas in Benin the cases were recorded at 6-wk intervals for 18 mo, so that the measured effect of the additional IRS may include a period when the insecticide, which is known to have a short residual duration, was no longer effective. In the Benin trial, LLINs were given only to target groups in the reference arm and in the study arm with IRS, whereas in the current trial ITNs were distributed to all age groups.

Large CRTs have recently been conducted in the Gambia [48],[49] and in Sudan [50] comparing villages with IRS and LLINs to villages with only LLINs, but the results have not yet been published.

Evidence of an added benefit from the combination intervention compared to IRS or ITNs alone has been shown in a number of observational studies [4],[45],[47],[51]–[55]. For example, children 2–14 y old consistently received added personal protection from using nets in addition to IRS on the island of Bioko, Equatorial Guinea (OR 0.71, 95% CI 0.59–0.86), and in Zambezia, Mozambique (OR 0.63, 95% CI 0.50–0.79) [4],[36]. In Pakistan, nets provided added protection against *P. vivax* and *P. falciparum* in refugee camps where IRS was conducted [56]. However, other studies observed no additional benefit from the combination compared to one intervention alone [46],[57],[58].

One interpretation of these divergent conclusions is that if the intervention present in both study arms is compromised or poorly implemented, the second method compensates for the deficiency of the first, providing apparent added protection that would otherwise not be seen. On the other hand, if the reference arm intervention is well implemented and efficacious in both study arms, there may be little or no scope for additional protection by a second intervention. ITN usage in the present trial was moderate, and hence the IRS protected many people who were not using a net in the ITN+IRS arm, whilst non-users in the ITN only arm remained unprotected. Any community or “mass effect” of ITNs on mosquito population size would have been limited because of the low community net usage. Therefore, the protective effect of ITNs in this study was possibly suboptimal. In Bioko, ITNs provided personal protection in the presence of IRS that was rendered only partially effective by moderate coverage (77%–79%) and use of an insecticide that did not outlast the long malaria season [36],[51]. Protopopoff et al. reported that in Burundi there was no additional reduction in infection prevalence in children from adding LLINs to IRS because high coverage (90%) of IRS had already reduced the sporozoite rate to a level where nets had no further impact [57]. In Sao Tome, where the IRS programme was poorly implemented, with low coverage and long intervals between spray rounds, there was an additional benefit from using ITNs and IRS compared to IRS alone [47]. However, on the neighbouring island of Principe, where IRS coverage was high (85%) and implemented on schedule, there was no added protection from ITNs in combination with IRS compared to IRS alone [46],[47].

Insecticide resistance may be another reason why differences have been seen for the effectiveness of the combination of IRS and ITNs, resulting in either an apparent “added” effect of the second effective intervention, if the first was ineffective due to insecticide resistance, or no added effect if the second intervention was ineffective due to insecticide resistance. In the study area of this trial, there was evidence for high levels of resistance to pyrethroids in *An. gambiae* s.s. The epidemiological impact of pyrethroid resistance on the effectiveness of ITNs is currently not known [59]. However, if the effectiveness of the ITNs was compromised [24] because of insecticide resistance, this would have enhanced our estimate of the additional benefit of non-pyrethroid IRS. If pyrethroid-treated nets were to be rendered partially ineffective in the presence of resistance, there would be a compelling case for combining ITNs with non-pyrethroid IRS.

An experimental hut trial in an area of Tanzania where the main vector is *An. arabiensis* found that if ITNs were used, the addition of IRS using insecticides with high irritancy such as dichlorodiphenyltrichloroethane (DDT) or lambda-cyhalothrin did not increase mosquito mortality or repel mosquitoes from the house [11]. However, the addition of IRS using pirimiphos-methyl, an organophosphate that has high toxicity and low irritancy, did increase mosquito mortality. These findings underscore that the interaction between the two interventions is complex and that the added protective effect will be dependent on the feeding and resting behaviours of particular malaria vectors, on the type of IRS insecticide used, on the susceptibility of local vectors to each of the insecticides in the combination, and on ITN usage [5]–[7],[11]. As a result, added protection may not be observed in all situations. A systematic review of all the trial results estimating the effectiveness of the combination of ITNs and IRS should be undertaken once the results of the trials in Sudan and the Gambia are available.

Nevertheless, this trial provides encouraging evidence for an additional benefit from applying IRS in combination with ITNs compared to ITNs alone. To our knowledge it is the first CRT to do so. The added protection from the supplementary use of IRS may in the case of bendiocarb be limited to only a few months, raising the question of whether residual insecticides of short duration are cost-effective when used in combination with ITNs. This study was conducted as an effectiveness study and not an efficacy study. The LLINs were distributed by a national UCC and therefore represented a real-life malaria control programme, including the challenges faced in achieving high coverage and usage of ITNs.

In conclusion, national malaria control programmes should consider implementing IRS in combination with ITNs if local ITN strategies alone are insufficiently effective and cannot be improved. A key consideration would be the additional cost of providing the combined intervention. Given the inconsistent trial evidence and the unproven generalisability of the findings of all studies that have investigated this question, it would be prudent for malaria control programmes implementing the two methods simultaneously to monitor the impact and cost-effectiveness of the combination to verify whether the additional resources have the desired effect.

## Supporting Information

Checklist S1 **CONSORT checklist.**
(DOCX)

Table S1 ***Pf*** **PR in children 0.5–14 y old in the ITN only and ITN+IRS arms (intention to treat) excluding the cluster that violated the protocol, in survey A, B, and C, Muleba District, Tanzania, 2012.**
(DOCX)

## Abbreviations

CRT: cluster randomised controlled trial
EIR: entomological inoculation rate
IRS: indoor residual spraying
ITN: insecticide-treated net
ITT: intention to treat
LLIN: long-lasting insecticidal net
OR: odds ratio
*Pf*PR: *Plasmodium falciparum* prevalence rate
PMI: President's Malaria Initiative
RDT: rapid diagnostic test
UCC: universal coverage campaign
WHO: World Health Organization
