# Supplementary material for: Indoor Residual Spraying in Combination with Insecticide-Treated Nets Compared to Insecticide-Treated Nets Alone for Protection against Malaria: A Cluster Randomised Trial in Tanzania
Source: PLoS Med. 2014 Apr 15;11(4):e1001630. doi: 10.1371/journal.pmed.1001630 (PMC3988001; doi:10.1371/journal.pmed.1001630)
Supplement: Table S1 — Pf PR in children 0.5–14 y old in the ITN only and ITN+IRS arms (intention to treat) excluding the cluster that violated the protocol, in survey A, B, and C, Muleba District, Tanzania, 2012. (DOCX) [file pmed.1001630.s002.docx]

Supplementary Table 1. *Pf*PR in children 0.5-14 years old in the ITN only and IRS+ITN arms (intention-to-treat) excluding the cluster that violated the protocol, in survey A, B and C, Muleba, Tanzania, 2012.

|  |  |  | **Prevalence** | **Odds ratio** |
| --- | --- | --- | --- | --- |
|  |  |  | %, [95% CI], (n) | OR, [95% CI], p=value |
|  | ***Survey A*** | Control | 23.4, [15.0,34.8], (2082) | 1.00 |
|  |  | Intervention | 13.6, [8.3,21.4], (2342) | 0.52, [0.24-1.12] p=0.093 |
|  | ***Survey B*** | Control | 31.7, [20.9,44.8], (1930) | 1.00 |
|  |  | Intervention | 12.7, [7.4,21.0], (2204) | 0.32, [0.14-0.72] p=0.007 |
|  | ***Survey C*** | Control | 25.5, [14.7,40.3], (1983) | 1.00 |
|  |  | Intervention | 13.4, [7.3,23.4], (2285) | 0.45, [0.17-1.19] p=0.106 |

**Note:** Survey A = 2 months after 1st intervention spray. Survey B = 6 months after 1^st^ intervention spray and 2 months after 2^nd^ spray. Survey C = 10 months after 1^st^ intervention spray and 6 months after 2^nd^ spray. OR = Odds ratio. CI = Confidence interval. n = Number tested. ^1^*Pf*PR = *Plasmodium falciparum* infection prevalence from RDTs.
